# Characterizing Behaviors That Influence the Implementation of Digital-Based Interventions in Health Care: Systematic Review

**DOI:** 10.2196/56711

**Published:** 2025-06-12

**Authors:** Sajan B Patel, Fahad M Iqbal, Kyle Lam, Amish Acharya, Hutan Ashrafian, Ara Darzi

**Affiliations:** 1 Department of Surgery and Cancer Imperial College London London United Kingdom; 2 Institute of Global Health Innovation Imperial College London London United Kingdom; 3 Medical Sciences Division University of Oxford Oxford United Kingdom

**Keywords:** human behaviors, behavior change, technology, intervention, eHealth, digital health

## Abstract

**Background:**

Successful implementation of any digital intervention in a health care setting requires adoption by all stakeholders. Appropriate consideration of behavioral change is a key driver that is often overlooked during implementation. The nonadoption, abandonment, scale-up, spread, and systems (NASSS) behavioral framework offers a broad evaluation of success for digital health solutions, and the theoretical domains framework (TDF) focuses particularly on adopters, identifying determinants of behavior and potential reasons for implementation issues.

**Objective:**

The aim of this study was to describe and characterize barriers and facilitators to the adoption of digital solutions within health care using behavioral frameworks: the NASSS and TDF.

**Methods:**

A systematic search was performed in 4 databases (ie, Ovid in MEDLINE, Embase, Health Management Information Consortium, and PsycINFO). Included studies reported a behavioral change by health care professionals following digital interventions or the practicality of delivering such interventions. Barriers and facilitators were identified, extracted, and classified using the NASSS framework and TDF. Risk of bias was assessed using the Mixed Methods Appraisal Tool.

**Results:**

The initial search result included 2704 unique studies, 12 of which met the inclusion criteria and from which data were extracted. All 12 scored ≥3 out of 5 stars on the Mixed Methods Appraisal Tool risk of bias assessment. Out of the 12 studies, 67% (n=8) were conducted in the United States, and 8% (n=1) each in India, Australia, the Netherlands, and Tanzania. The NASSS framework identified facilitators and barriers in 4 domains: the condition or illness, technology, value proposition, and adopter system. The TDF framework identified 8 relevant domains, including knowledge, skills, and beliefs about capabilities. Key facilitators included intuitive technology design aligned with existing workflows, clear communication of value propositions to adopters, adequate provision of training resources tailored to adopters’ knowledge levels, and ensuring organizational readiness for technological change. Conversely, significant barriers involved disruptions to clinical workflow, inadequate adopter training or confidence levels, unclear value propositions leading to disengagement, insufficient consideration of cognitive load impacts, such as alert fatigue, and limited organizational preparedness. Notably, psychological factors such as optimism, intentions, and social influences were underreported.

**Conclusions:**

This study delineated and analyzed various critical behavioral factors impacting the adoption and implementation of digital interventions in health care. Based on these findings, future research must consider the key factors reported and alternative approaches to assess behaviors influencing adoption that are not presented in the current scientific literature.

**Trial Registration:**

PROSPERO CRD42022264937; https://www.crd.york.ac.uk/PROSPERO/view/CRD42022264937

## Introduction

### Background

The successful implementation of digital interventions in health care settings critically depends on their adoption by various stakeholders and health care team members. This process is often hindered by an inadequate consideration of the necessary behavioral changes during the implementation phase. To effectively support these changes, a thorough understanding of the influencing factors is essential. Frameworks, such as the nonadoption, abandonment, scale-up, spread, and systems (NASSS) approach, offer a broad evaluation of the success of digital health solutions [1,2]. Previous reviews have used the NASSS framework to assess contextual factors influencing implementation success of online consultations in primary care [3] and computerized clinical decision support systems in hospitals, identifying adopters as a key determinant [4].

The theoretical domains framework (TDF) provides a theory-informed approach to identify determinants of behavior and potential reasons for implementation issues, focusing particularly on the adopters [5]. Ignoring these behavioral influences can result in poor individual uptake, unsuccessful implementation on both local and systemic levels, or limited growth and innovation of the intervention. This aspect is particularly overlooked when introducing novel remote monitoring technologies [6].

In the neurorehabilitation setting, clinician attitudes and digital literacy, training availability, organizational readiness and support, perceived usefulness, perceived ease of use, and patient engagement have been identified as critical determinants of technology uptake [7,8]. While these studies provide valuable insights into specific clinical contexts, they focus primarily on clinical and organizational aspects of digital health implementation. Our systematic review builds upon this foundation by integrating 2 complementary behavioral frameworks, NASSS and TDF, to comprehensively characterize barriers and facilitators across diverse health care settings. This approach allows for a broader understanding of the complex interplay between technological, organizational, and behavioral factors influencing digital health adoption, offering a more nuanced perspective on how to enhance implementation strategies across different health care environments.

Health care digitization is an increasingly important field and is crucial to meet the evolving demands of health care, as described in the Topol Review by Health Education England [9]. This review forecasts substantially impacts from technological advancements such as telemedicine, smartphone apps, and diagnostic sensors and wearables. The COVID-19 pandemic further accelerated changes in health care delivery, exemplified by the adoption of technology-enabled virtual wards allowing patients to receive care at home instead of in the hospital [10]. The implementation of innovative wearable sensing solutions in the UK National Health Service has encountered obstacles, particularly the lack of consideration for human behavior, which significantly affects the potential of the technology and patient safety [11]. Moreover, engaging key stakeholders is often overlooked during the digital solutions implementation process [12].

### Objectives

The aim of this study was to describe and characterize barriers and facilitators to the adoption and implementation of digital solutions within health care using behavioral frameworks.

## Methods

### Study Design

This systematic review was conducted in accordance with the PRISMA (Preferred Reporting Items for Systematic Reviews and Meta-Analyses) guidelines, which is an evidence-based minimum set of items for reporting systematic reviews and meta-analyses [13].

### Registration and Protocol

The review was registered on PROSPERO (CRD42022264937).

### Research Question

This review sought to answer the following question: Which human behaviors influence the adoption and implementation of digital-based interventions in health care?

### Search Strategy and Databases

A systematic search with expert librarian support was performed using the electronic databases MEDLINE, Embase, Health Management Information Consortium, and PsycINFO, for papers published in the English language. The search was conducted using a list of terms relating to human behaviors and digital tools in health care; the complete search strategy is available in Multimedia Appendix 1.

All identified studies were uploaded to Covidence (Veritas Health Innovation), a Cochrane-supported systematic review package tool [14]. Initial title and abstract screening were conducted by 2 investigators (SBP and FMI) to determine if the eligibility criteria were met. Discrepancies were resolved by discussion. Studies meeting the inclusion criteria underwent full-text screening (SBP and FMI); further studies not captured by the search were identified through bibliometric cross-referencing of reference lists.

### Study Selection Criteria and Outcome Measures

Eligibility criteria were developed for title and abstract and refined full-text screening, and the final search was performed on July 13 2021. Included studies either reported outcomes relating to a change in behavior or practice of health professionals, or the practicalities of delivering such interventions using technological supports. Full inclusion and exclusion criteria are outlined in Textbox 1.

**Inclusion and exclusion criteria for this systematic review.**

**Inclusion criteria**

Article type: Peer-reviewed articles reporting digital health interventions with full texts available

Language: English language only

Population: Health care professionals or staff

Outcomes: Either reported outcomes relating to change in behavior or practice of health care professionals, or the practicalities of delivering such interventions using technological supports

**Exclusion criteria**

Article type: Conference proceedings, dissertations, books, abstracts

Language: Non-English languages

Population: Non–health care staff populations

Outcomes: Outcomes unrelated to behavior or practicalities

### Data Extraction

For all included studies, barriers and facilitators were independently identified, extracted, and classified by 2 investigators (SBP and FMI) using 1 or more of the 7 NASSS framework domains: the condition or illness, the technology, the value proposition, the adopter system, the organization, the wider (institutional and societal) context, and the interaction and mutual adaptation between all these domains over time [1]. All extracted data were then further organized using the NASSS framework subcategories with the exception of the adopter system, which was further classified using the 14 domains of the TDF, allowing for a comprehensive framework for examining implementation factors [5] by SBP and FMI. These included knowledge, skills, social or professional role and identity, belief about the capabilities, optimism, reinforcement, intentions, goals, memory, attention, and decision processes, environmental context and resources, social influences, emotion, and behavioral regulation. Where studies were independently reviewed by SBP and FMI, consensus was achieved through discussion of any discrepancies.

### Quality Assessment (Risk of Bias)

Risk of bias was assessed using the Mixed Methods Appraisal Tool (MMAT) [15]. This is a critical appraisal tool designed for systematic reviews, including qualitative, quantitative, and mixed methods studies. It consists of frameworks for 5 categories of studies; qualitative research, randomized controlled trials, nonrandomized studies, quantitative descriptive studies, and mixed methods studies. Quality assessment was performed by one reviewer (SBP) and validated by a second reviewer (FMI), independently.

### Data Synthesis

Once the data had been extracted into the individual NASSS and TDF headings, 2 authors (SBP and FMI) reviewed and discussed each heading and subheading and synthesized the findings from the included studies to answer the review question. All studies with an MMAT score of ≥3 stars were included in the discussion and with specific risk of bias scores being considered in the discussion and synthesis.

## Results

### Overall Study Characteristics

**Figure 1:**
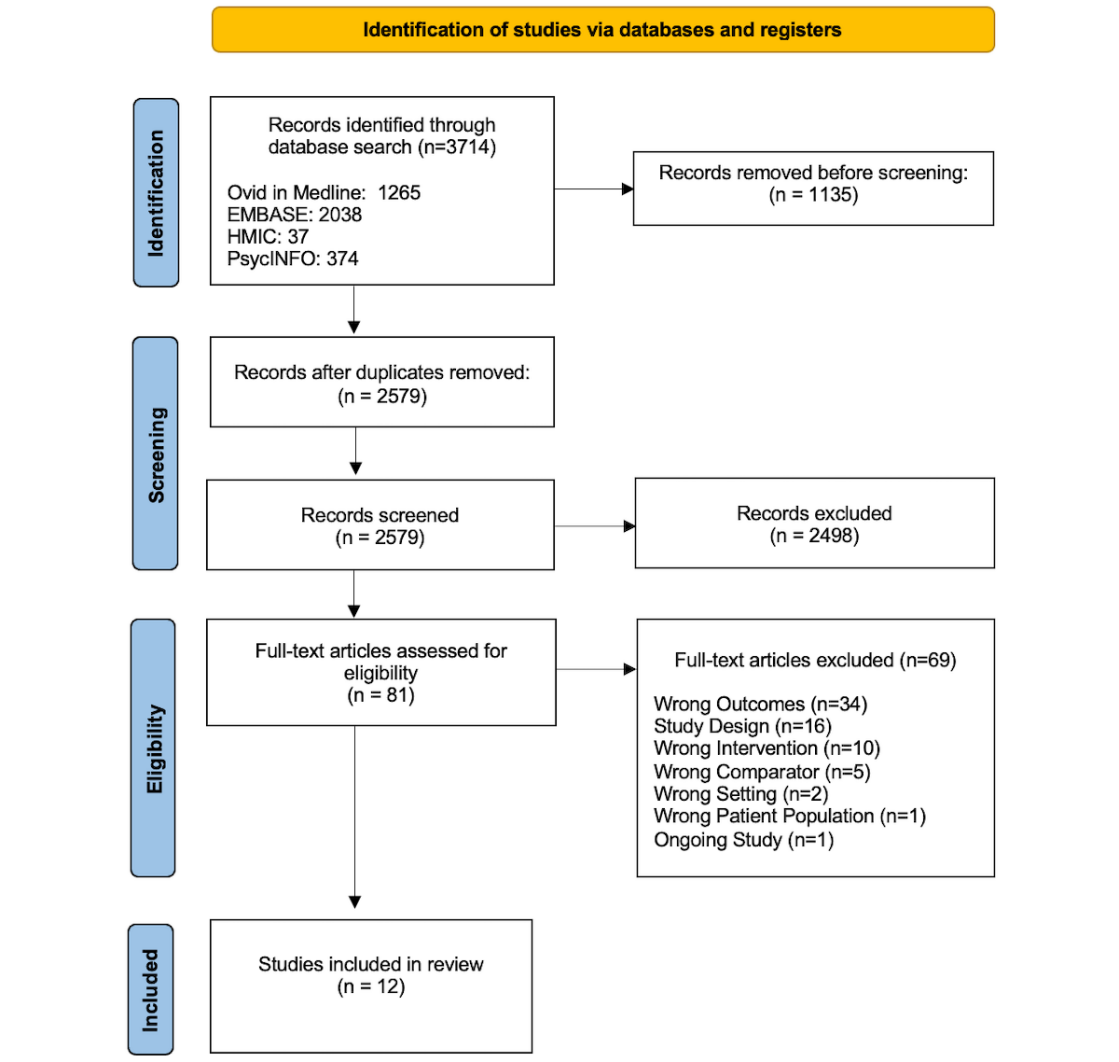
Search and study selection process for this review.

A total of 2704 unique citations were retrieved through literature searches. A full-text review was performed for 81 articles, with 12 meeting the inclusion criteria for analysis. A PRISMA flow diagram is provided in Figure 1. Table 1 summarizes the characteristics of the included studies. Of the 12 included studies, there were 4 (33%) qualitative studies, 2 (17%) quantitative randomized controlled trials, 3 (25%) quantitative nonrandomized studies, and 3 (25%) mixed methods studies. Eight studies were carried out in the United States, and one each in India, Australia, the Netherlands, and Tanzania.

**Table 1:** Characteristics of included studies with quality score (Mixed Methods Appraisal Tool) [15].

Study, year	Title	Journal	Study design	Country	Quality^a^
Curran et al [16], 2020	Integrated displays to improve chronic disease management in ambulatory care: A SMART on FHIR application informed by mixed methods user testing	*Journal of the American Medical Informatics Association*	Mixed methods approach—sequential explanatory design	United States	*****
Doyle et al [17], 2012	Computers in the examination room and the electronic health record: physicians’ perceived impact on clinical encounters before and after full installation and implementation	*Family Practice*	Qualitative research	United States	***
Kaphle et al [18], 2015	Adoption and usage of mHealth technology on quality and experience of care provided by frontline workers: observations from rural India	*JMIR mHealth and uHealth*	Cohort study	India	***
Mann et al [19], 2020	Impact of clinical decision support on antibiotic prescribing for acute respiratory infections: a cluster randomized implementation trial	*Journal of General Internal Medicine*	Randomized controlled trial	United States	***
Montoy et al [20], 2020	Association of default electronic medical record settings with health care professional patterns of opioid prescribing in emergency departments	*JAMA Internal Medicine*	Quality improvement	United States	*****
North et al [21], 2019	Impact of human factors testing on medical device design: validation of an automated CGM sensor applicator	*Journal of Diabetes Science and Technology*	Comparative ease of use analysis	United States	***
Page et al [22], 2019	Selection and use of decision support alerts in electronic medication management systems in Australian hospitals: a survey of implementers	*Journal of Pharmacy Practice and Research*	Cross-sectional study	Australia	***
Pamplin et al [23], 2020	Improving clinician decisions and communication in critical care using novel information technology	*Military Medicine*	Usability+validation	United States	***
Scheepers-Hoeks et al [24], 2013	Physicians’ responses to clinical decision support on an intensive care unit: comparison of four different alerting methods	*Artificial Intelligence in Medicine*	Randomized controlled trial	The Netherlands	***
Shield et al [25], 2010	Gradual electronic health record implementation: new insights on physician and patient adaptation	*Annals of Family Medicine*	Mixed methods	United States	****
van Pelt et al [26], 2021	The development of an electronic clinical decision and support system to improve the quality of antenatal care in rural Tanzania: lessons learned using intervention mapping	*Frontiers of Public Health*	Qualitative research	Tanzania	****
Weingart et al [27], 2014	Do drug interaction alerts between a chemotherapy order-entry system and an electronic medical record affect clinician behavior?	*Journal of Oncology Pharmacy Practice*	Cross-sectional study	United States	*****

^a^The total number of MMAT criteria that are scored as yes.

Table 2 displays a summary of included study designs, limitations, and conclusions. These were heterogeneous in terms of the intervention being assessed (eg, electronic health care records, alert systems, clinical decision support tools, mobile health platforms) and the adopters using the technology (general practitioners, specialist doctors, physician assistants, pharmacists, nurses, midwives, and administrative staff).

**Table 2:** A summary of included studies.

Study	Aim of the study	Technology used	Inclusion criteria	Participants (n)	Description	Limitations	Conclusions
Curran et al [16]	Development and evaluation of novel EHR^a^ integrated display add-on for COPD^b^	EHR integrated display add-on application	Faculty providers in UUH^c^ Division of Family Medicine or primary care providers in UUH Community Clinics	13	Clinical measures (proportion of recommended tasks completed and total clinical tasks completed) Efficiency measures (recommended tasks completed per min, total mouse clicks, and total keyboard clicks) Participant reported workload measures (NASA-TLX^d^) Key themes from user feedback interviews	Single center with single EHR focusing on standardized COPD case scenarios with many assumptions, limiting real world generalizability. Sample size was small for quantitative analysis.	Use of the add-on was linked to more recommended COPD care being carried out than without. Add-on users also required fewer keystrokes per task.
Doyle et al [17]	Comparing physicians’ perspectives before and after EHR implementation	EHR	—^e^	24	Perceived fear over patient privacy and confidentiality Variability in physician skills using EHR and actual difficulty learning the new system Perceived negative effects on quality of patient care, perceived and actual improvements in accessing data and medication management, and prevention of potential medico-legal issues Concerns around potential impact on physician-patient interaction Actual improvement in the ability to make shared patient decisions	Interviews conducted shortly after installation; therefore the novelty of computers in exam rooms may influence outcomes	When physicians and staff positively anticipate benefits of EHR use in the examination rooms, they demonstrate a willingness to work through their initial fears and concerns to adapt to the new EHR. Physicians using the EHR on computers in the examination rooms appear to take on a role that fosters a more collaborative physician-patient relationship. They perceive themselves to assume a teaching role, searching the internet for medical information and making joint health care decisions and treatment plans with patients. This collaboration may foster higher engagement of patients regarding follow-up with their own treatments.
Kaphle et al [18]	Develop a framework to assess whether mHealth^f^ platforms affect the quality and experience of care provided by staff	CommCare—mHealth app for maternal and newborn care	ASHAs^g^	14 home visits included in final analysis; 13 ASHAs with differing levels of CommCare adoption; 3 ASHAs not using CommCare	User characteristics: literacy levels, education levels, previous mobile experience, and age Quality score made up of: use of mHealth app, proficiency in using the app, quality of home visits, experience of home visits, and duration of visits Technology adoption: number of form submissions in the last 30, 60, or 90 days	Underpowered as it only compared mHealth versus no mHealth app rather than quality and experience of care qualitatively. Social and programming factors that can affect technology adoption and quality and experience of care directly and via the technology were not analyzed. Observing ASHAs’ home visits can affect their performance, leading to biased indicators.	Two levels of CommCare adoption were found: in the first midwives (ASHAs) were still learning the design and content of the technology and used it as a tool for reporting, whereas in the second they were more proficient in using CommCare, understood how the tool is designed, and used it appropriately as a job aid for reporting, as well as for counseling during home visits. The first level had lower quality and experience of home visits versus the second. Higher levels of CommCare adoption were significantly associated with higher quality and experience of care. Individual characteristics: illiteracy did impact the adoption and quality of home visits, whereas education, previous mobile experience did not affect
Mann et al [19]	Assess clinical decision support on antibiotic-prescribing rates for respiratory infections in primary care settings	iCPR^h^ in EHRs	Internal medicine+family medicine primary care practices within 2 large academic health systems	33 practices; 541 providers; 100,573 patient visits	No difference in antibiotic prescription rates, biochemical or radiological orders, or total inappropriate antibiotic prescribing between control and intervention groups	Adaptive trial design in academic adult centers only, limiting generalizability	A CDS^i^ tool did not significantly reduce antibiotic prescribing for respiratory infections. Failure to change provider behaviors was likely driven by low usage rates of the CDS tool; affected by system factors such as alert fatigue and complexity of EHR workflows, patient factors such as patient expectations for antibiotics, and multiple provider factors.
Montoy et al [20]	Effect of change in EHR default settings on opioid prescribing in ED^j^	Changing EHR default settings for opioid prescriptions	HCPs^k^, physicians, nurse practitioners, physician assistants) working in study EDs	104 ED HCPs; 4320 prescriptions for study opioids in final sample	Number of opioid tablets prescribed at ED discharge, distribution of quantity prescribed, proportion of prescriptions from >12 tablets under each default setting, and proportion of prescriptions written for the default quantity	Limited generalizability to non-ED HCPs or other centers and limited insight into detailed description of opioid prescribing (eg, choice of opioid)	A reduction in the quantities of opioids prescribed through an EHR add-on
North et al [21]	Ease of new automated sensor applicator compared to the previous manual application	“One button” automated sensor applicator for use with Dexcom g6 system	—	8	Evaluation of each sensor’s installation steps on their perception, cognition, and action requirements (1-5) to evaluate users’ ability to perceive important inputs from the device or instructions, understand information and perform the appropriate actions. Ease of use for inserting the sensor and for attaching the transmitter (1-10)	Small sample size. Did not involve patients so their perceptions are unknown	Using a human factors engineering approach in the design of rtCGM^l^ systems increases patient safety, supports adherence in device use, and enhances clinician effectiveness and efficiency in patient training. Our study demonstrates that effective end-user analysis and early identification of design issues in the formative development phase and iterative design changes resulted in a sensor application device that is easy to use and that may enhance frequent and persistent rtCGM use in a broad population of potential users
Page et al [22]	Determining alert thresholds for electronic medication management systems	EMM^m^ systems	Direct involvement with the implementation of an EMM system in an Australian public or private hospital or have knowledge of their hospital’s configuration decisions, evaluation, and governance	15	—	Total 15 respondents representing only 26 Australian hospitals. Limited to mainly pharmacists, not all HCPs	Alert categories are used across the Australian hospital system for drug-drug interactions among others. Starting with small well-designed alerts was a key lesson learnt. Evaluations at a local level have been limited due to stakeholders being less convinced about benefits in a local setting.
Pamplin et al [23]	Clinician perceptions of an EHR system	Add-on app (novel health information technology software) for 16-bed burns ICU^n^	—	—	—	Single center study with small sample size limiting generalizability of findings. Limited time using the IT systems due to the nature of the simulated patients’ conditions made it difficult to assess the experience	Following minimal training, clinicians effectively used the novel IT while performing realistic patient care for highly complex, simulated patients. For similarly complex care scenarios, a less experienced team performed similarly to a more experienced team. In both studies, clinicians expressed preference for the add-on, particularly its messaging support and data organization or trend visualizations
Scheepers-Hoeks et al [24]	To determine the effect of 4 different alert presentation methods on alert compliance after the implementation of an advanced CDSS^o^ on the ICU in our hospital	CDSS 4 groups: pharmacy intervention, physician alert list, EHR section, pop-up alert	—	3281 patients; 384 with alerts. User satisfaction survey: 10 physicians; 6 responses	Alert compliance, user satisfaction	Physician group changed frequently in the study period. Limited to ICU environment and 1 center, Randomization method (based on patient number) did not distribute patients equally over the 4 alert methods	In this study, a routine advanced CDSS with a set of 13 locally developed clinical rules was implemented in an ICU. We showed that the alert presentation method used was associated with the compliance percentage of the clinical rules in the CDSS, and therefore, with the potential effectiveness of the system. This study showed that active alerts such as pop-ups and pharmacy intervention were more effective than passive alerts, which do not automatically appear within the clinical workflow. Furthermore, pop-up alerts and pharmacy intervention were the most preferred method by clinicians in this study. More research is required to extend these results to other departments and other hospitals, as well as to other types of CDSS and different types of alerting
Shield et al [25]	Effectiveness of EHR implementation for patients and staff	EHR	English speaking, aged ≥18 years , visiting for an acute problem or follow-up	13 faculty physicians; 13 residents; 170 clinical encounters; 20 family care center staff in focus groups;	Patients and staff perceptions of technology generally in favor for documentation, workflow, and patient safety. Concerns over doctors being a barrier to implementation, impacts on patient-physician relationship and confidentiality. Less time spent out of the room with EHRs	Participating patients may have been more satisfied with the physicians than those who declined. Conducting exit interviews in the clinic may have inhibited patient criticism	Strong patient trust in the physician-patient relationship was maintained and workflow improved with EHR implementation. Gradual EHR implementation may help support the development of beneficial physician and staff adaptations, while maintaining positive patient-physician relationships and fostering the sharing of medical information
van Pelt et al [26]	Development and implementation of an app based electronic clinical decision and support system	Nurse assistant app	—	2 interviewees	Qualitative evaluation from documents describing development and implementation of the app and semi structured interviews aligned with this	Researchers involved in evaluation were involved in implementation risk of bias. Only 2 members of the team interviewed due to the inability to contact	Future program developers to engage the community and listen to their insights, focus on clear program goals and the desired change, consult or involve a behavior change specialist, and anticipate potential problems in unexpected circumstances
Weingart et al [27]	Changes in clinician behavior in drug prescribing from an add-on app	Add-on to the chemotherapy entry system that alerts prescribers about potential drug interactions	—	3.5 million orders in COE^p^ for chemotherapy drugs and supportive care medication. A total of 29,592 COE-LMR^q^ drug interaction alerts were studied	Intention to monitor patients, adjust dose, or acknowledge that the patient already tolerated the drug combination were common reasons for dismissing the alerts Alerts caused a change in prescriber behavior by 13% to change or cancel drug orders	Small number of practice sites and single COE system and EHR	Patients are at risk of serious interactions among drugs ordered for cancer care and those provided for nononcology medical care. Organizations that lack an integrated system of chemotherapy ordering and prescription writing, those that do not routinely reconcile the ambulatory medication list, and those that rely on paper-based chemotherapy orders should develop countermeasures to identify and prevent potentially serious drug interactions.

^a^EHR: electronic health record.

^b^COPD: chronic obstructive pulmonary disease.

^c^UUH: University of Utah Health.

^d^NASA-TLX: NASA Task Load Index.

^e^Not available.

^f^mHealth: mobile health.

^g^ASHA: accredited social health activist.

^h^iCPR: integrated clinical prediction rules.

^i^CDS: clinical decision support.

^j^ED: emergency department.

^k^HCP: health care provider.

^l^rtCGM: real-time continuous glucose monitor.

^m^EMM: electronic medications management.

^n^ICU: intensive care unit.

^o^CDSS: clinical decision support system.

^p^COE: chemotherapy order system.

^q^LMR: longitudinal medical record.

### Barriers and Facilitators

#### NASSS Framework

##### Overview

**Figure 2:**
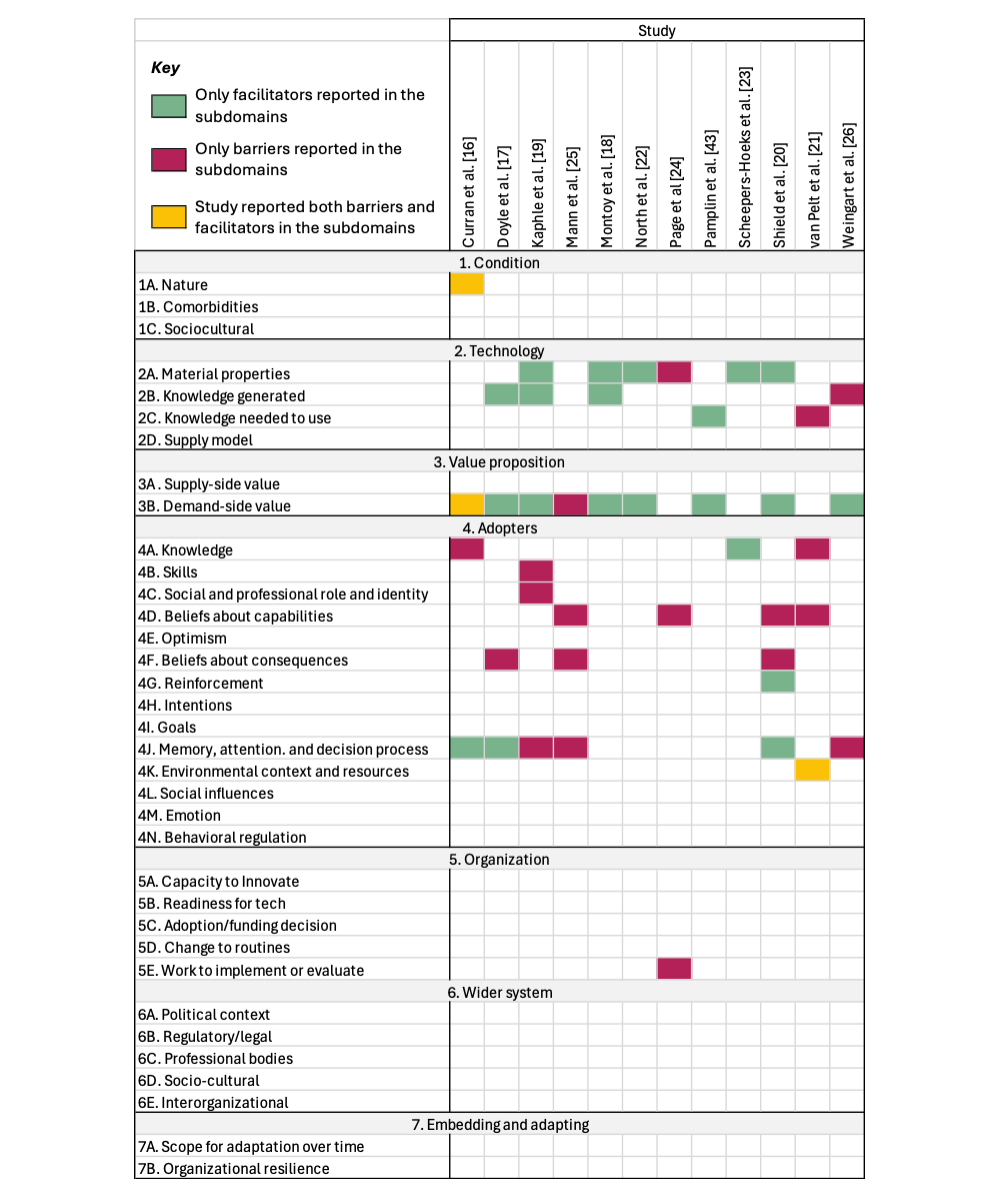
Behaviors acting as barriers and facilitators to the implementation of digital-based interventions in health care from included studies mapped to nonadoption, abandonment, scale-up, spread, and systems approach (NASSS) and theoretical domains framework (TDF) domains and subdomains by study. Matrix demonstrating frequency and distribution of barriers and facilitators reported in each study across NASSS domains (1-7) and subdomains. For the domain adopters, the subdomains comprise of TDF domains (4A-4N).

Figure 2 [1,5,16-27] shows a breakdown by studies into domains of the NASSS framework and subdomains of the TDF. Specifically, 4 NASSS domains emerged as dual influencers, acting both as facilitators and barriers: the nature of the condition or illness, the characteristics of the technology itself, the value proposition of the technology, and the system of adopters. In contrast, 1 domain was exclusively identified as a barrier: the organizational context. Notably, 2 domains did not appear in the included studies: its broader contextual impact of the technology and the process of its embedding and adaptation over time.

##### The Condition or Illness

The nature of the condition or illness plays a dual role in the implementation of digital health interventions, reported in one study as acting as both a facilitator and a barrier. For example, Curran et al [16] demonstrated that the standardized parameters and management approaches of chronic obstructive pulmonary disease facilitated the integration of an electronic health record add-on. This add-on aimed to optimize care delivery. However, challenges arose when participants were required to input binary responses about symptoms, such as the presence or absence of dyspnea. Participants found this oversimplified, as determining breathing status often involves nuanced judgments beyond a simple binary choice and subsequently underreported dyspnea.

##### The Technology

Digital interventions’ success is heavily influenced by their technological aspects, both regarding the software’s features and the hardware used. Ten studies reported these, highlighting features such as accessible patient information, drug interaction alerts, and educational tools that enable physicians to better engage and inform patients [17,18,20]. Transitioning from paper to electronic records has demonstrated improvements in legibility, accuracy, and record management, which incentivizes users to drive implementation [25]. However, technology that disrupted user workflow or failed to align with clinical practice was shown to be a significant barrier. Issues such as insensitive clinical alerts, overriding of chemotherapy drug interaction alerts, and the occurrence of device failures and technical bugs have been noted as impediments causing users to disengage with the technology [26]. Ease of use and the availability of instructional materials are crucial for successful implementation and user adoption [21].

##### The Value Proposition

The value proposition of a technology is divided into supply-side value: the business case for the developers and demand-side value—the desirability, efficacy, safety, and cost-effectiveness from the users’ perspective. The included studies did not explicitly address the supply-side value. Nine of the 12 included studies reported the demand-side value, frequently reporting the desirability and efficacy as an outcome measure of the study. In all instances, these are addressed under other domains. Few studies reported safety, and none explicitly addressed cost-effectiveness.

###### The Adopter System

The adopter system significantly influences the implementation of digital interventions in health care. Nine of the 12 included studies reported the adopter system as a facilitator or barrier, which are further categorized and mapped into 8 out of the 14 domains of the TDF.

###### Knowledge

Effective implementation hinges on understanding and tailoring interventions to the adopters’ knowledge. Three studies reported knowledge as a barrier or facilitator. Pharmacists trained in receiving drug alerts evaluated recommendations and consulted with doctors when necessary [24]. However, variability in clinical judgment, such as assessing dyspnea, introduces complexity in decision-making [16]. The importance of consistently training all staff was evident when individuals missed a 2-day training event, highlighting the challenge of ensuring all adopters have a uniform level of knowledge [26].

###### Skills

Skills were reported as a barrier in one study. Adopters’ literacy levels significantly affect the success of digital interventions. Higher literacy levels among adopters led to better experiences during home visits with a mobile health app for maternal and newborn care [18].

###### Social or Professional Role and Identity

Differences in adopter demographics impacted the perceived quality of digital interventions as reported by one study. Older adult adopters reported lower quality experiences compared to younger adults when using a mobile health app for maternal and newborn care [18].

###### Beliefs About Capabilities

Four included studies reported that adopters’ beliefs in their capabilities influence the success of digital interventions. Health care professionals sometimes felt they did not need additional support or doubted their ability to use new tools effectively. This led to disregard for clinical decision support tools and skepticism about the sensitivity and specificity of electronic systems [22]. In addition, concerns about inadequate typing skills among nurses’ aides affected the transition to electronic health records [25]. Finally, health care workers would mask true opinions when faced with problem solving, an imperative eventuality with novel implementation [26].

###### Beliefs About Consequences

Three included studies reported beliefs about consequences as a barrier. Adopters’ concerns about the implications of digital interventions included data security [17,19,25], the impact on physician-patient relationships [17], and potential increases in workload [25].

###### Reinforcement

One study reported reinforcement as a barrier with physicians initially hesitant to use electronic health records growing more confident over time. Strategies developed to maintain patient engagement during consultations included verbal cues and strategic pausing [25].

###### Memory, Attention, and Decision Processes

Six studies reported memory, attention, and decision processes as either a facilitator or barrier. Digital interventions that reduce the cognitive load, such as electronic health records, facilitated smoother consultations and allowed health care professionals to focus more on patients [16,17,25]. However, some interventions led to increased attention on task completion, detracting from patient interaction [18]. Alert fatigue from excessive or irrelevant alerts also hindered the effectiveness of alert-based systems [19,27].

###### Environmental Context and Resources

One study reported the environmental context and resources as both a barrier and facilitator. The availability of resources such as free electronic devices can influence overall engagement and behavior [26]. Integrating interventions with existing technological infrastructures is crucial for a better fit within digital health care systems.

###### The Organization

One study discussed the need to test their alert system at a lead site before the roll out to others, which offers a potential barrier [22]. The included studies did not explore the context of the organizations they worked in, how ready organizations were for technology-supported change, the adoption, the funding decision processes, and its impact on behavior.

#### Risk of Bias Assessment

Multimedia Appendix 2 [16-27] provides a detailed breakdown of the methodological quality criteria from the MMAT for each study. All 12 studies reviewed scored ≥3 out of 5 stars, with only 2 (17%) satisfying all the quality criteria. Notably, 11 studies presented clear research questions and used appropriate data collection methods to address these questions effectively.

## Discussion

### Principal Findings

This systematic review aimed to characterize barriers and facilitators influencing the adoption of digital interventions in health care using 2 established behavioral frameworks: the NASSS and TDF. Twelve studies met the inclusion criteria; analysis identified 5 NASSS domains (“condition or illness,” “technology,” “value proposition,” “adopter system,” and “organization”) and 8 TDF domains (“knowledge,” “skills,” “social or professional role,” “beliefs about capabilities,” “beliefs about consequences,” “reinforcement,” “memory, attention, or decision processes,” and “environmental context/resources”). Our findings highlight that successful implementation critically depends on aligning interventions with adopters’ capabilities and workflows, ensuring intuitive technology design, clearly communicating value propositions, providing adequate training resources, and addressing organizational readiness.

Our findings showed the digital intervention itself is a pivotal factor in the success of adoption, particularly when it enhances the clinician’s experience and aligns with existing workflows. Intrusive interventions can disrupt these workflows, leading to reduced engagement by users. The risks associated with software and hardware failures, identified as significant barriers to adoption, must be carefully considered during the design and delivery phases. One study [28] highlighted technical factors, including network issues and a lack of infrastructure, as common barriers, while formal instructions or guidance can mitigate some of these challenges [29]. Where users are unable to recognize the value proposition or experience negative effects from the technology, they may lose interest in adoption of the technology.

Several psychological and social factors were underreported and are critical in understanding the adoption of digital health care interventions, in keeping with previous findings identifying motivation to change and willingness to adopt novel approaches as important but underexplored psychological factors [8]. TDF domains such as optimism, intentions, goals, and behavioral regulation were not reported in included studies or the wider literature, which could be likely due to digital health implementation studies focusing on final evaluation. Emotions such as fear have been described as a barrier; however, it was also not identified in the included studies [28].

This review identified the capabilities, beliefs, and workflows of adopters in the clinical environment as crucial for the successful implementation of digital interventions. Factors, such as knowledge, skills, social and professional roles, and identity are paramount. For example, ensuring adopters have the necessary knowledge and literacy, both basic and digital, to use the digital intervention effectively is vital [30]. Personal and psychological barriers, including age and education levels, have been described as multifaceted [28,30]. In the wider literature, user-friendly design and intuitive navigation are also often cited as facilitators for ease of use [7,8,31].

Our findings indicated that adopters’ beliefs about their capabilities and the consequences of using digital interventions, such as concerns over patient information security, play a significant role. The integration of digital interventions into existing workflows requires a balance between capabilities and cognitive load. Cognitive burdens are a recognized barrier to the implementation of digital health interventions [31], specifically regarding situational awareness and decision-making impairment [32]. The literature suggests a need to adapt consultation skills to accommodate digital interventions, especially in the context of increased remote consultations following the COVID-19 pandemic [33,34]. However, there is limited research into communication skills required while adopting digital health interventions in person. Disruptions, such as alert fatigue from excessive notifications, are recognized barriers and contribute to resistance to engagement with digital interventions [35-37].

Adoption and implementation are significantly influenced by the nature of the intervention. Indeed, factors facilitating or hindering the implementation of an electronic health record system likely differ from those affecting an integrated alert system. As illustrated in one study [25], most facilitators and barriers identified during the initial implementation of Electronic Health Record Systems relate more to the broader concept of health care digitization than to the specifics of an individual digital intervention [38]. Moreover, the scope of digital interventions examined in the included studies was limited, often excluding technologies such as automated laboratory or radiology reports, communication systems, and cloud services. Furthermore, the existing literature tends to inadequately detail the implementation strategies for these digital interventions, with a focus that shifts from the design process to the achieved outcomes [28].

Despite these variations, there are common threads, such as the critical importance of adopter engagement and training. However, it is essential to note the inherent limitations of the TDF and NASSS frameworks used in these studies. The TDF, while comprehensive, can be complex and cumbersome to apply, potentially overlooking the dynamic and context-specific nature of behavior. The NASSS framework may oversimplify the system-level interactions and changes over time, potentially failing to capture the nuanced and evolving nature of technology implementation in health care settings. Our use of the NASSS framework does not incorporate the associated complexity categorization tools, which would offer a useful scope for future work. Recognizing these limitations is crucial in understanding the context and implications of the review’s findings and in guiding future research and implementation strategies.

Further research to answer several important questions is required. First, the included studies overlooked several domains, including those within the NASSS framework: “the wider system” and “embedding and adaptation over time,” as well as within the TDF: “optimism,” “intentions,” “goals,” “social influences,” “emotion,” and “behavioral regulation.” While not commonly reported in scientific literature unless directly related to study outcomes, these factors are likely critical in the implementation of digital health care interventions and warrant further investigation through qualitative methods that explore these elements to deepen our understanding of the value proposition and other overlooked areas.

Second, academic research and quality improvement in health care often emphasize predetermined protocols and outcomes, neglecting the iterative nature of development and implementation. This could potentially be driven by limited funding opportunities for long-term funding continuing beyond the evaluation phase [4]. Adopting a more flexible approach, similar to start-up methodologies, could enhance learning and integration within health care settings. This approach would include a focus on adopter customer discovery, rapid hypothesis testing, iterative design, and continuous maintenance of digital health solutions, important when integrating novel digital solutions in complex organizations [39,40]. Use of an implementation model, for example, the exploration, preparation, implementation, sustainment framework [41], which describes 4 well-defined phases that align with and guide the implementation process, may offer a useful next step for digital innovators in the implementation stage. Another important consideration beyond implementation is considering service design to help teams execute more successful digital health solution adoption projects [40].

Policy makers should integrate human factors and behavioral evaluations into the implementation of digital solutions to modify workflows effectively. Such integration is crucial for comprehending the financial and technical prerequisites for successful adoption. Collaboration among all key stakeholders, including patients, health care professionals, policy makers, and industry, is essential.

In addition, there should be a strategic allocation of resources, encompassing dedicated training and support for health care staff. This approach is vital to ensure that variations in digital literacy are considered, aiming to cultivate a digitally proficient workforce. NHS England’s commitment of GDP £200 (USD $265) million in funding, with an additional GDP £250 (USD $331) million in 2023 and 2024, underscores the importance of these initiatives [42,43]. It is imperative that these funds are strategically allocated to address the issues highlighted, fostering a more effective and responsive digital health care system.

### Limitations and Strengths

Several limitations must be acknowledged in interpreting our findings. Considerable heterogeneity was observed among included studies regarding intervention types, settings, and methodologies. This limits direct comparisons and generalizability of specific barriers and facilitators across different technological interventions or clinical contexts. This study was limited to scientific articles and did not include white papers, governmental or organizational reports, or blog posts; these articles could be considered in the future and may provide details relating to NASSS and TDF domains, particularly in the development process. Furthermore, limited qualitative data available restricted a deeper exploration into psychological or social influences on adopter behavior. Finally, this review highlighted the paucity of published literature as multiple factors within the frameworks were devoid of any literature; as a result, this affected the number of meaningful conclusions that could be drawn, and our findings show the starting points when it comes to implementing digital solutions in health care.

Despite these limitations, this review has several key strengths. By integrating 2 established behavioral frameworks, NASSS and TDF, we provided a comprehensive characterization of behaviors influencing digital health adoption that complements existing literature. Unlike previous reviews that primarily focused on clinical or organizational factors alone [7], our approach incorporates adopter-level psychological determinants, such as beliefs about capabilities and consequences, memory processes, attention demands, and environmental contexts. Finally, by explicitly identifying gaps in current reporting practices regarding overlooked behavioral domains (eg, optimism or behavioral regulation), we offer clear directions for future research to deepen understanding of human behaviors critical for successful digital intervention implementation across health care settings.

### Conclusions

In conclusion, our systematic review identified critical behavioral domains influencing digital health intervention adoption across diverse health care settings using the NASSS framework and TDF. Key facilitators include intuitive technology design aligned with existing workflows, clear communication of value propositions to adopters, adequate provision of training resources tailored to adopters’ knowledge levels, and ensuring organizational readiness for technological change. Conversely, significant barriers include disruptions to clinical workflow, inadequate adopter training or confidence levels, unclear value propositions leading to disengagement, insufficient consideration of cognitive load impacts, such as alert fatigue, and limited organizational preparedness. Stakeholders aiming for successful implementation must strategically address these identified behavioral factors through targeted interventions such as user-centered design processes, tailored educational programs for clinicians with varying digital literacy levels, proactive management of cognitive burdens associated with new technologies, and fostering organizational cultures supportive of technological innovation. Recognizing and addressing factors that are not commonly reported in academic literature is vital for developing robust frameworks that can guide the successful implementation of digital interventions in future health care settings.

## Data Availability

All data presented in this study were extracted from published original data. The data that support the findings of this study are available from the corresponding author upon reasonable request.

## Appendix

Multimedia Appendix 1 Search strategies.

Multimedia Appendix 2 Risk of bias assessment of included studies using the mixed methods appraisal tool.

Multimedia Appendix 3 PRISMA checklist.

## Abbreviations

MMAT: Mixed Methods Appraisal Tool
NASSS: nonadoption, abandonment, scale-up, spread, and systems
PRISMA: Preferred Reporting Items for Systematic Reviews and Meta-Analyses
TDF: theoretical domains framework
