# Supplementary material for: Characterizing Behaviors That Influence the Implementation of Digital-Based Interventions in Health Care: Systematic Review
Source: J Med Internet Res. 2025 Jun 12;27:e56711. doi: 10.2196/56711 (PMC12203025; doi:10.2196/56711)
Supplement: Multimedia Appendix 1 [file jmir_v27i1e56711_app1.docx]

# Supplementary Tables

Supplementary Table 1A – Search Strategies - Ovid MEDLINE^Ò^

|  | **Ovid MEDLINE(R) ALL<1946 to July 12, 2021>** | **Results per line** |
| --- | --- | --- |
|  | **Date: 13/07/2021** |  |
| 1 | exp Health Personnel/ | 546951 |
| 2 | "attitude of health personnel"/ or attitude to computers/ | 129645 |
| 3 | (staff or health professional$ or health care worker$ or health worker$ or doctor$ or nurse$ or general practitioner$).mp. | 785211 |
| 4 | or/1-3 | 1112544 |
| 5 | exp Telemedicine/ | 35489 |
| 6 | (digital health or DHIs).mp. [mp=title, abstract, original title, name of substance word, subject heading word, floating sub-heading word, keyword heading word, organism supplementary concept word, protocol supplementary concept word, rare disease supplementary concept word, unique identifier, synonyms] | 3417 |
| 7 | (ehealth or e-health or electronic health or mobile health or mhealth or m-health or mobile phone$ or smartphone$ or smart-phone$ or smart technolog$ or smart device$ or cell$ phone$ or social media or wearables or machine wearable or machine wearable device$ or  wearable technol$ or activity track$).mp. | 103614 |
| 8 | ((wearable or smart) adj3 device$).mp. | 9714 |
| 9 | (telehealth or tele-health or telemedicine or tele-medicine or telerehabilitation or tele-rehabilitation or tele consultation or tele- consultation or m-mental or e-mental or emental or digital mental or electronic mental).mp. | 40915 |
| 10 | ((internet or computer or online or virtual$) adj2 (deliver$ or via)).mp. | 11133 |
| 11 | cell phone/ | 9151 |
| 12 | Mobile Applications/ | 8163 |
| 13 | (Mobile Application$ or mobile app or mobile apps).mp. [mp=title, abstract, original title, name of substance word, subject heading word, floating sub-heading word, keyword heading word, organism supplementary concept word, protocol supplementary concept word, rare disease supplementary concept word, unique identifier, synonyms] | 11958 |
| 14 | ((webbased or web-based or website$ or the web) adj2 (deliver$ or via)).mp. | 2745 |
| 15 | Therapy, Computer-Assisted/ | 6911 |
| 16 | computer-assisted therap$.mp. | 99 |
| 17 | or/5-16 | 167618 |
| 18 | 4 and 17 | 27915 |
| 19 | (intervention$ or program$ or education$ or solution$ or therap$ or treat$).mp. | 11831405 |
| 20 | 18 and 19 | 17791 |
| 21 | (human factor$ or human issues or human elements or human needs or human behavio$ or behaviours or behaviors).mp. | 285940 |
| 22 | (individual$1 adj2 (behavio$ or performance or factors or needs or issues)).mp. | 37540 |
| 23 | *Behavior/ | 14141 |
| 24 | (behavio$ adj2 change$).mp. | 52134 |
| 25 | ((staff or health professional$ or health care worker$ or health worker$ or doctor$ or nurse$ or general practitioner$) adj2 (factor$ or issue$ or needs or behavio$)).mp. | 10300 |
| 26 | (human adj2 interact$).mp. [mp=title, abstract, original title, name of substance word, subject heading word, floating sub-heading word, keyword heading word, organism supplementary concept word, protocol supplementary concept word, rare disease supplementary  concept word, unique identifier, synonyms] | 15250 |
| 27 | or/21-26 | 391866 |
| 28 | 20 and 27 | 1279 |
| 29 | limit 28 to English language | 1265 |

Supplementary Table 2 – Search Strategies - Embase^Ò^

|  | **Embase <1974 to 2021 July 12>** | **Results per line** |
| --- | --- | --- |
|  | **Date: 13/07/2021** |  |
| 1 | exp health care personnel/ | 1680367 |
| 2 | exp health personnel attitude/ | 190988 |
| 3 | attitude to computers/ | 3407 |
| 4 | (staff or health professional$ or health care worker$ or health worker$ or doctor$ or nurse$ or general practitioner$).mp. | 1105459 |
| 5 | or/1-4 | 2257937 |
| 6 | exp telemedicine/ | 50872 |
| 7 | (digital health or DHIs).mp. [mp=title, abstract, heading word, drug trade name, original title, device manufacturer, drug manufacturer, device trade name, keyword, floating subheading word, candidate term word] | 3718 |
| 8 | (ehealth or e-health or electronic health or mobile health or mhealth or m-health or mobile phone$ or smartphone$ or smart-phone$ or smart technolog$ or smart device$ or cell$ phone$ or social media or wearables or machine wearable or machine wearable device$ or  wearable technol$ or activity track$).mp. | 129009 |
| 9 | ((wearable or smart) adj3 device$).mp. | 7522 |
| 10 | (telehealth or tele-health or telemedicine or tele-medicine or telerehabilitation or tele-rehabilitation or tele consultation or tele- consultation or m-mental or e-mental or emental or digital mental or electronic mental).mp. | 51004 |
| 11 | ((internet or computer or online or virtual$) adj2 (deliver$ or via)).mp. | 15404 |
| 12 | mobile phone/ | 18566 |
| 13 | smartphone/ | 16756 |
| 14 | mobile application/ or mobile health application/ | 16297 |
| 15 | (Mobile Application$ or mobile app or mobile apps).mp. [mp=title, abstract, heading word, drug trade name, original title, device manufacturer, drug manufacturer, device trade name, keyword, floating subheading word, candidate term word] | 16481 |
| 16 | ((webbased or web-based or website$ or the web) adj2 (deliver$ or via)).mp. | 3973 |
| 17 | exp computer assisted therapy/ | 15360 |
| 18 | computer-assisted therap$.mp. | 4844 |
| 19 | or/6-18 | 224462 |
| 20 | 5 and 19 | 64692 |
| 21 | (intervention$ or program$ or education$ or solution$ or therap$ or treat$).mp. | 15845199 |
| 22 | 20 and 21 | 44139 |
| 23 | (human factor$ or human issues or human elements or human needs or human behavio$ or behaviours or behaviors).mp. | 340319 |
| 24 | (individual$1 adj2 (behavio$ or performance or factors or needs or issues)).mp. | 47407 |
| 25 | (behavio$ adj2 change$).mp. | 83143 |
| 26 | *behavior/ | 62433 |
| 27 | ((staff or health professional$ or health care worker$ or health worker$ or doctor$ or nurse$ or general practitioner$) adj2 (factor$ or issue$ or needs or behavio$)).mp. | 12472 |
| 28 | ((online or internet or social media) adj2 behav$).mp. [mp=title, abstract, heading word, drug trade name, original title, device manufacturer, drug manufacturer, device trade name, keyword,  floating subheading word, candidate term word] | 2051 |
| 29 | (human adj2 interact$).mp. [mp=title, abstract, heading word, drug trade name, original title, device manufacturer, drug manufacturer, device trade name, keyword, floating subheading word, candidate term word] | 22914 |
| 30 | or/23-29 | 530945 |
| 31 | 22 and 30 | 2977 |
| 32 | conference.pt. | 4899031 |
| 33 | 31 not 32 | 2058 |
| 34 | limit 33 to english language | 2038 |

Supplementary Table 1B – Search Strategies - APA PsycINFO

|  | **APA PsycINFO <1987 to July Week 1 2021 >** | **Results per line** |
| --- | --- | --- |
|  | **Date: 13/07/2021** |  |
| 1 | exp health personnel/ | 153330 |
| 2 | health personnel attitudes/ | 20032 |
| 3 | (staff or health professional$ or health care worker$ or health worker$ or doctor$ or nurse$ or general practitioner$).mp. | 203006 |
| 4 | or/1-3 | 294150 |
| 5 | exp electronic health services/ or digital interventions/ or mobile health/ or exp telemedicine/ or wearable devices/ | 12321 |
| 6 | (digital health or DHIs).mp. [mp=title, abstract, heading word, table of contents, key concepts, original title, tests & measures, mesh] | 469 |
| 7 | (ehealth or e-health or electronic health or mobile health or mhealth or m-health or mobile phone$ or smartphone$ or smart-phone$ or smart technolog$ or smart device$ or cell$ phone$ or social media or wearables or machine wearable or machine wearable device$ or wearable technol$ or activity track$).mp. | 34580 |
| 8 | ((wearable or smart) adj3 device$).mp. | 922 |
| 9 | (telehealth or tele-health or telemedicine or tele-medicine or telerehabilitation or tele-rehabilitation or tele consultation or tele- consultation or m-mental or e-mental or emental or digital mental or electronic mental).mp. | 8598 |
| 10 | ((internet or computer or online or virtual$) adj2 (deliver$ or via)).mp. | 7305 |
| 11 | mobile phones/ or smartphones/ | 6001 |
| 12 | mobile applications/ | 1270 |
| 13 | (Mobile Application$ or mobile app or mobile apps).mp. [mp=title,  abstract, heading word, table of contents, key concepts, original title, tests & measures, mesh] | 2870 |
| 14 | ((webbased or web-based or website$ or the web) adj2 (deliver$ or via)).mp. | 1027 |
| 15 | computer assisted therapy/ | 1149 |
| 16 | computer-assisted therap$.mp. | 1193 |
| 17 | or/5-16 | 53919 |
| 18 | 4 and 17 | 6030 |
| 19 | (intervention$ or program$ or education$ or solution$ or therap$ or treat$).mp. | 1712878 |
| 20 | 18 and 19 | 4438 |
| 21 | (human factor$ or human issues or human elements or human needs or human behavio$ or behaviours or behaviors).mp. | 265004 |
| 22 | (individual$1 adj2 (behavio$ or performance or factors or needs or issues)).mp. | 30425 |
| 23 | *behavior/ | 12574 |
| 24 | (behavio$ adj2 change$).mp. | 36453 |
| 25 | ((staff or health professionals or health care worker$ or health worker$ or doctor$ or nurse$ or general practitioner$) adj2 (factor$ or issue$ or needs or behavio$)).mp. | 5102 |
| 26 | (human adj2 interact$).mp. [mp=title, abstract, heading word, table of contents, key concepts, original title, tests & measures, mesh] | 17710 |
| 27 | or/21-26 | 340623 |
| 28 | 20 and 27 | 522 |
| 29 | limit 28 to english language | 501 |
| 30 | dissertation abstract.pt. | 389360 |
| 31 | book.pt. | 392261 |
| 32 | or/30-31 | 781621 |
| 33 | 29 not 32 | 374 |

Supplementary Table 1D – Search Strategies - HMIC

|  | **HMIC <1979 to May 2021>** | **Results per line** |
| --- | --- | --- |
|  | **Date: 13/07/2021** |  |
| 1 | exp health service staff/ | 55365 |
| 2 | staff attitudes/ | 568 |
| 3 | (staff or health professional$ or health care worker$ or health worker$ or doctor$ or nurse$ or general practitioner$).mp. | 94630 |
| 4 | or/1-3 | 103087 |
| 5 | telemedicine/ | 1332 |
| 6 | (digital health or DHIs).mp. [mp=title, other title, abstract, heading words] | 100 |
| 7 | (ehealth or e-health or electronic health or mobile health or mhealth or m-health or mobile phone$ or smartphone$ or smart-phone$ or smart technolog$ or smart device$ or cell$ phone$ or social media or wearables or machine wearable or machine wearable device$ or wearable technol$ or activity track$).mp. | 1414 |
| 8 | ((wearable or smart) adj3 device$).mp. | 13 |
| 9 | telehealth/ | 545 |
| 10 | (telehealth or tele-health or telemedicine or tele-medicine or telerehabilitation or tele-rehabilitation or tele consultation or tele- consultation or m-mental or e-mental or emental or digital mental or electronic mental).mp. | 2027 |
| 11 | ((internet or computer or online or virtual$) adj2 (deliver$ or via)).mp. | 255 |
| 12 | mobile telephones/ | 280 |
| 13 | (Mobile Application$ or mobile app or mobile apps).mp. [mp=title, other title, abstract, heading words] | 32 |
| 14 | ((webbased or web-based or website$ or the web) adj2 (deliver$ or via)).mp. | 65 |
| 15 | computer-assisted therap$.mp. | 3 |
| 16 | or/5-15 | 3633 |
| 17 | 4 and 16 | 1127 |
| 18 | (intervention$ or program$ or education$ or solution$ or therap$ or treat$).mp. | 105085 |
| 19 | 17 and 18 | 501 |
| 20 | (human factor$ or human issues or human elements or human needs or human behavio$ or behaviours or behaviors).mp. | 3963 |
| 21 | (individual$1 adj2 (behavio$ or performance or factors or needs or issues)).mp. | 1571 |
| 22 | (behavio$ adj2 change$).mp. | 1763 |
| 23 | ((staff or health professional$ or health care worker$ or health worker$ or doctor$ or nurse$ or general practitioner$) adj2 (factor$ or issue$ or needs or behavio$)).mp. | 1464 |
| 24 | (human adj2 interact$).mp. [mp=title, other title, abstract, heading words] | 43 |
| 25 | or/20-24 | 8145 |
| 26 | 19 and 25 | 37 |
| 27 | limit 26 to English | 37 |
