# Supplementary material for: Characterizing Behaviors That Influence the Implementation of Digital-Based Interventions in Health Care: Systematic Review
Source: J Med Internet Res. 2025 Jun 12;27:e56711. doi: 10.2196/56711 (PMC12203025; doi:10.2196/56711)
Supplement: Multimedia Appendix 2 [file jmir_v27i1e56711_app2.pdf]

Risk of bias assessment of included studies using the mixed methods appraisal tool [15].

Study	Screening		Qualitative					Quantitative randomized controlled trials					Quantitative nonrandomized					Mixed methods					Score[a]
	S1[b]	S2[c]	1.1[d]	1.2[e]	1.3[f]	1.4[g]	1.5[h]	2.1[i]	2.2[j]	2.3[k]	2.4[l]	2.5[m]	3.1[n]	3.2[o]	3.3[p]	3.4[q]	3.5[r]	5.1[s]	5.2[t]	5.3[u]	5.4[v]	5.5[w]	
Doyle et al [17]	N[x]	C[y]	C	C	Y[z]	Y	Y	—[aa]	—	—	—	—	—	—	—	—	—	—	—	—	—	—	***
van Pelt et al [21]	Y	Y	Y	N	Y	Y	Y	—	—	—	—	—	—	—	—	—	—	—	—	—	—	—	****
Kaphle et al [19]	Y	Y	Y	N	Y	Y	N	—	—	—	—	—	—	—	—	—	—	—	—	—	—	—	***
Page et al [24]	Y	Y	Y	Y	Y	Y	Y	—	—	—	—	—	—	—	—	—	—	—	—	—	—	—	*****
Scheepers-Hoeks et al [23]	Y	Y	—	—	—	—	—	Y	N	Y	C	Y	—	—	—	—	—	—	—	—	—	—	***
Mann et	Y	Y	—	—	—	—	—	Y	N	Y	C	Y	—	—	—	—	—	—	—	—	—	—	***

al [25]																							
Montoy et al [18]	Y	Y	—	—	—	—	—	—	—	—	—	—	Y	Y	Y	Y	Y	—	—	—	—	—	*****
North et al [22]	Y	Y	—	—	—	—	—	—	—	—	—	—	N	Y	Y	N	Y	—	—	—	—	—	***
Weingart et al [26]	Y	Y	—	—	—	—	—	—	—	—	—	—	Y	Y	Y	Y	Y	—	—	—	—	—	*****
Pamplin et al [43]	Y	Y	—	—	—	—	—	—	—	—	—	—	—	—	—	—	—	N	Y	Y	Y	C	***
Curran et al [16]	Y	Y	—	—	—	—	—	—	—	—	—	—	—	—	—	—	—	Y	Y	Y	Y	Y	*****
Shield et al [20]	Y	Y	—	—	—	—	—	—	—	—	—	—	—	—	—	—	—	Y	Y	Y	N	Y	****

[a]Score refers to the total number of criteria that are scored yes. Numbers refer to MMAT methodological quality criteria.

[b]S1 (screening question 1): are there clear research questions?

[c]S2 (screening question 2): do the collected data allow to address the research?

[d]1.1: is the qualitative approach appropriate to answer the research question?

[e]1.2: are the qualitative data collection methods adequate to address the research question?

[f]1.3: are the findings adequately derived from the data?

[g]1.4: is the interpretation of results sufficiently substantiated by data?

[h]1.5: is there coherence between qualitative data sources, collection, analysis, and interpretation?

[i]2.1: is randomization appropriately performed?

[j]2.2: are the groups comparable at baseline?

[k]2.3: are there complete outcome data?

[l]2.4: are outcome assessors blinded to the intervention provided?

[m]2.5: did the participants adhere to the assigned intervention?

[n]3.1: are the participants representative of the target population?

[o]3.2: are measurements appropriate regarding both the outcome and intervention (or exposure)?

[p]3.3: are there complete outcome data?

[q]3.4: are the confounders accounted for in the design and analysis?

[r]3.5: during the study period, is the intervention administered (or exposure occurred) as intended?

[s]5.1: is there an adequate rationale for using a mixed methods design to address the research question?

[t]5.2: are the different components of the study effectively integrated to answer the research question?

[u]5.3: are the outputs of the integration of qualitative and quantitative components adequately interpreted?

[v]5.4: are divergences and inconsistencies between quantitative and qualitative results adequately addressed?

[w]5.5: do the different components of the study adhere to the quality criteria of each tradition of the methods involved?

[x]N: no.

[y]C: cannot tell.

[z]Y: yes.

[aa]Not available.
